# Prevalence of residual left ventricular structural changes after one year of antihypertensive treatment in patients of African descent: role of 24-hour pulse pressure

**DOI:** 10.5830/CVJA-2012-001

**Published:** 2012-04

**Authors:** Elena N Libhaber, Mohammed R Essop, Carlos D Libhaber, Geoffrey P Candy, Elena N Libhaber, Gavin R Norton, Angela J Woodiwiss, Pinhas Sareli

**Affiliations:** Department of Cardiology, University of the Witwatersrand, and Chris Hani Baragwanath Hospital, Johannesburg, South Africa; Department of Cardiology, University of the Witwatersrand, and Chris Hani Baragwanath Hospital, Johannesburg, South Africa; Department of Nuclear Medicine, University of the Witwatersrand, Johannesburg, South Africa; Department of Surgery, University of the Witwatersrand, Johannesburg, South Africa; Cardiovascular Pathophysiology and Genomics Research Unit, School of Physiology, Faculty of Health Sciences, University of the Witwatersrand, Johannesburg, South Africa; Cardiovascular Pathophysiology and Genomics Research Unit, School of Physiology, Faculty of Health Sciences, University of the Witwatersrand, Johannesburg, South Africa; Cardiovascular Pathophysiology and Genomics Research Unit, School of Physiology, Faculty of Health Sciences, University of the Witwatersrand, Johannesburg, South Africa; Cardiovascular Pathophysiology and Genomics Research Unit, School of Physiology, Faculty of Health Sciences, University of the Witwatersrand, Johannesburg, South Africa

**Keywords:** left ventricular geometry, antihypertensive therapy, ambulatory blood pressure, pulse pressure

## Abstract

**Objectives:**

One year of antihypertensive therapy may normalise left ventricular (LV) structure in 51% of hypertensive patients of European descent. Whether similar effects can be achieved in patients of African descent, who have a high prevalence of concentric LV hypertrophy (LVH) and remodelling, is unknown.

**Methods:**

In 103 hypertensive patients in the Baragwanath Hypertension study we evaluated the prevalence of residual LV structural changes (echocardiography) after four and 13 months of stepwise antihypertensive therapy.

**Results:**

After 13 months of therapy, 24-hour blood pressure control was achieved in 47% of patients. At baseline, 51.5% of patients had concentric LVH, 19% eccentric LVH and 12% concentric LV remodelling. Despite changes in LV mass index (*p* < 0.01) and relative wall thickness (*p* < 0.05) with treatment, the proportion of patients with a normal LV mass or geometry increased only from 17.5 to 25% (*p* > 0.05), while 26% remained with concentric LVH (*p* < 0.001 compared to baseline), 25% with eccentric LVH and 23% with concentric LV remodelling (*p* < 0.05 compared to baseline). Residual structural changes were associated with 24-hour pulse pressure (*p* = 0.02), but not with 24-hour systolic or diastolic blood pressure or clinic blood pressure.

**Conclusions:**

Even after a year of antihypertensive therapy, a high proportion (74%) of hypertensives of African ancestry retained residual LV structural changes, an effect that was associated with 24-hour pulse pressure but not systolic or diastolic blood pressures or clinic blood pressure in this ethnic group.

## Abstract

Left ventricular hypertrophy (LVH) is an established independent predictor of morbidity and mortality.^1^-^3^ However, the geometry of the heart in hypertensive LVH is a heterogeneous change. Some patients develop a concentric pattern where wall thickness increases out of proportion to chamber diameter, while others develop eccentric LVH where wall thickness increases in parallel with chamber diameters.^4^ The possibility that LV geometric patterns may refine the ability to predict cardiovascular events beyond LVH was suggested two decades ago,^5^ and more recent evidence provides substantial support for this notion in hypertensives^6^,^7^ in the general population^8^ following myocardial infarction,^9^ in diabetes mellitus,^10^ and in those with a normal LV ejection fraction.^11^,^12^ Therefore an important goal of therapy in hypertensives and other patient groups should be to reduce the prevalence of concentric LVH, eccentric LVH and LV remodelling.

Although some^13^-^18^ but not all^19^,^20^ studies have demonstrated that antihypertensive therapy decreases both LVH and relative wall thickness, these studies were largely conducted in ethnic groups with a low prevalence of concentric LVH at baseline (6–30%).^11^,^13^-^15^ Those studies reporting on the prevalence of concentric LVH and remodelling before and after drug therapy suggest that after therapy, six to 16% of patients may have concentric LVH and only a small proportion, concentric LV remodelling.^13^-^15^ Whether the same low residual prevalence rates of concentric LVH or remodelling remain after drug therapy in patient populations with a high prevalence of concentric LVH or remodelling at baseline is unknown. In this regard it is well recognised that LV relative wall thickness is higher in patients of African ancestry than in other patient populations.^21^ Therefore, in the present study, we aimed to identify the ability of 13 months of antihypertensive therapy to normalise LV structure in hypertensives of African ancestry and the blood pressure parameter most closely associated with residual LV structural abnormalities.

## Methods

The protocol was approved by the University of the Witwatersrand Committee for Research in Human Subjects (approval number: M940106). The Baragwanath Hypertension Study was a single-centre, randomised, open-label trial conducted at the Chris Hani-Baragwanath Hospital from 1994 to 1997. The enrolment criteria have previously been described.^22^,^23^ Briefly, men and women of African descent were enrolled if they were 18 to 70 years of age and free of clinically significant cardiovascular or non-cardiovascular disorders. All patients gave informed, written consent.

Patients diagnosed as being hypertensive after a two-week placebo run-in period were randomised if in addition, their daytime diastolic blood pressure was 90 to 114 mmHg on ambulatory monitoring. Eligible patients were randomised to receive therapy as previously described.^23^ Patients were followed up at monthly intervals and if at the first monthly follow-up visit the target blood pressure was not reached, the daily dose of the first-line drug was increased. At two months, patients who had still not achieved target blood pressure values received additional antihypertensive agents and at a further follow-up visit, daily doses were either increased or additional therapy added.^23^

All patients randomised in the Baragwanath trial underwent echocardiography at baseline. Patients were followed up for 13 months. However, only patients in whom high-quality echocardiograms could be obtained were eligible for inclusion in the follow-up echocardiographic sub-study. Of the 233 patients included in the sub-study, data on 103 were available at 13 months. No significant differences were found in the baseline characteristics between the 103 patients included in the sub-study and the non-participants, except for body mass index (BMI), which was higher in the participants (31.1 ± 6.0 kg/m^2^) than in the non-participants (29.5 ± 5.9 kg/m^2^, *p* = 0.04).

At baseline and at follow-up visits, nurse-derived clinic blood pressure was assessed after the patient had rested in the sitting position for 10 minutes. Measurements were obtained three times, consecutively, according to guidelines. The same nurse performed the clinic blood pressure readings in all patients. Pulse pressure was calculated as differences between systolic and diastolic blood pressure. Furthermore, oscillometric SpaceLabs (model 90207) devices were programmed to obtain blood pressure readings every 15 minutes from 06:00 to 22:00 and every 30 minutes from 22:00 to 06:00. The intra-individual blood pressure means were weighted by the time interval between successive blood pressure readings.^23^ Twenty-four-hour blood pressure control was defined as mean values of < 130/80 mmHg, and clinic blood pressure control as values of < 140/90 mmHg.

At randomisation, four months and 13 months, two-dimensional targeted M-mode (short-axis view) echocardiograms were obtained with a Hewlett-Packard Sonos 2500 system using a 2.5-MHz transducer and analysed according to the American Society of Echocardiography Convention.^24^ All measurements were recorded on videotape and analysed by the same experienced echocardiographer who was unaware of the clinical data of the patients. Replicate measurements of LVM index showed that in the present study population, the inter-observer and intra-observer coefficients of variation were 12.4 and 11.4%, respectively.

Left ventricular mass (LVM) was determined as previously described and indexed (LVMI) to height^2.7^. Left ventricular end-diastolic mean wall thickness (MWT) was calculated from (LV end-diastolic septal wall thickness + LV end-diastolic posterior wall thickness)/2. Left ventricular end-diastolic relative wall thickness (RWT) was calculated from (LV end-diastolic septal wall thickness + LV end-diastolic posterior wall thickness)/ LV end-diastolic diameter. Left ventricular hypertrophy (LVH) was defined as an LVMI > 51 g/m^2.7^ in both women and men.^25^

Concentric LVH was defined as the presence of LVH and a RWT > 0.45. Eccentric LVH was defined as the presence of LVH and a RWT ≤ 0.45. Concentric remodelling was defined as the presence of a RWT > 0.45, but without LVH. Normal LV geometry was considered as the absence of LVH and a RWT ≤ 0.45. An abnormal LV geometry was considered as the presence of concentric LVH, eccentric LVH and or concentric LV remodelling.

## Statistical analysis

Database management and statistical analyses were performed with SAS software, version 9.13 (SAS Institute Inc, Cary, NC, USA). Data are expressed as mean ± SD. Differences in continuous variables between the patients assigned to each of the four LV geometric patterns were identified on an ANOVA, followed by the test of Scheffe, or the Kruskal-Wallis test when the distribution was abnormal.

To assess changes in continuous variables from zero to four and 13 months of treatment, a repeated-measurements ANOVA was performed. To compare the prevalence rates of the four patterns of LV geometry at zero and 13 months of therapy, a Chi-square test followed by Bonferroni correction was performed. To evaluate longitudinal changes in the prevalence rates of the four LV geometric patterns, a Mantel-Haenszel strategy for repeated measurements (according to the SAS program) or a McNemar test was performed. To identify predictors of abnormal geometry, a stepwise multiple logistic regression model was used, where age, gender, BMI and blood pressure were included in the model. A *p* < 0.05 was considered to be statistically significant.

## Results

Table 1 shows the characteristics at baseline of patients grouped according to the mass and geometry of the left ventricle. At baseline, 51.5% of patients had concentric LVH, while 19% had eccentric LVH and 12% concentric LV remodelling. Therefore, only 17.5% of the patients at baseline had a normal left ventricle. The age and gender distribution across the four groups was similar. However, patients with concentric LVH had a higher BMI and off-treatment 24-hour clinic systolic blood pressure (BP) and pulse pressure (PP). Patients with eccentric LVH had a higher BMI.

**Table 1:** Baseline Characteristics Of Patients Grouped According To Left Ventricular Structure

	*Geometry*			
*Geometry*	*Normal*	*Concentric LVH*	*Concentric remodelling*	*Eccentric LVH*
Number (%)	18 (17.5)	53 (51.5)**	12 (12)	20 (19)
BMI (kg/m^2^)	28.6 ± 7.8	32.4 ± 5.1**	28.0 ± 3.1	31.7 ± 6.7**
Age (years)	51.9 ± 9.7	52.4 ± 9.2	48.3 ± 10.2	50.1 ± 10.3
Female gender [*n* (%)]	14 (78)	44 (83)	10 (83)	17 (85)
24-hour SBP (mmHg)	146 ± 11	158 ± 16**	148 ± 20	147 ± 11
24-hour DBP (mmHg)	96 ± 7	98 ± 6	94 ± 8	96 ± 7
Clinic SBP (mmHg)	164 ± 19	178 ± 20*	161 ± 25	168 ± 17
Clinic DBP (mmHg)	99 ± 2	104 ± 9	100 ± 9	101 ± 9
Clinic PP (mmHg)	65.7 ± 16.1	73.5 ± 16.7**	60.2 ± 20.0	67.6 ± 15.1
24-hour PP (mmHg)	50.5 ± 8.9	59.4 ± 11.8*	53.1 ± 15.3	52.1 ± 9.5
LVM (g)	151 ± 29	248 ± 62**	159 ± 32	222 ± 36**
LVMI (g/m^2.7^)	42.8 ± 8.8	74.3 ± 16.5**	44.0 ± 3.4	66.0 ± 11.7**
LV mean wall thickness (mm)	9.4 ± 1.2	11.4 ± 1.6**	11.5 ± 1.4**	11.0 ± 1.1**
LV relative wall thickness (ratio)	0.38 ± 0.06	0.56 ± 0.10	0.56 ± 0.10	0.39 ± 0.04**

BMI, body mass index; LV, left ventricular; LVH, left ventricular hypertrophy; SBP, systolic blood pressure; DBP, diastolic BP; PP, pulse pressure; LVM, left ventricular mass; LVMI, left ventricular mass index. *p < 0.05, **p < 0.01 for comparisons between the four left ventricular geometric patterns.

Table 2 shows mean BP values and the percentage BP control at baseline and after four and 13 months of antihypertensive treatment. Over the four-month treatment period, 46% of patients achieved clinic BP values of < 140/90 mmHg, and by 13 months of therapy, 64% had clinic BP values that were < 140/90 mmHg. Over the four-month treatment period, 46% of patients achieved 24-hour BP values of < 130/80 mmHg and by 13 months of therapy 47% had 24-hour BP values that were < 130/80 mmHg.

**Table 2:** Blood Pressure And Echocardiographic Values At Baseline And After Four And 13 Months Of Treatment (*n* = 103)

	*Baseline*	*4 months*	*13 months*
Clinic SBP/DBP (mmHg)	171 ± 21/102 ± 10	148 ± 21/91 ± 11**	135 ± 19/85 ± 10**
Clinic heart rate (beats/min)	71 ± 10	72 ± 10	72 ± 11
24-hour SBP/DBP (mmHg)	152 ± 16/97 ± 7	129 ± 15/87 ± 9**	127 ± 12/80 ± 8**
24-hour heart rate (beats/min)	77 ± 9	76 ± 10	75 ± 10
Clinic BP control^†^ [*n* (%)]	0	47 (46)	66 (64)
24-hour BP control^†^ [*n* (%)]	0	47 (46)	48 (47)
Clinic PP (mmHg)	69.5 ± 17.1	56.7 ± 15.3**	49.8 ± 14.6**
24-hour PP (mmHg)	55.7 ± 11.9	46.0 ± 9.8**	46.3 ± 8.7**
LV end-diastolic diameter (mm)	46.6 ± 5.8	45.3 ± 5.3*	45.2 ± 5.1*
LV end-systolic diameter (mm)	30.1 ± 5.6	29.0 ± 5.0**	28.6 ± 5.4**
LV septal wall thickness (mm)	12.5 ± 2.1	11.4 ± 1.9**	11.4 ± 1.9**
LV posterior wall thickness (mm)	11.3 ± 2.2	10.5 ± 1.7**	10.4 ± 1.8**
LV mean wall thickness (mm)	11.9 ± 2.0	11.0 ± 1.5**	10.9 ± 1.5**
LV relative wall thickness (ratio)	0.50 ± 0.11	0.47 ± 0.10*	0.47 ± 0.11*
LVM (g)	215 ± 64	182 ± 48**	179 ± 43**
LVMI (g/m^2.7^)	63.7 ± 19.0	53.7 ± 13.9**	52.8 ± 12.3**
LVH [*n* (%)]	73 (71)	48 (47)**	52 (50)**
LV relative wall thickness [*n* (%)]	65 (63)	55 (53)*	50 (49)**

LV, left ventricular; LVH, left ventricular hypertrophy; SBP, systolic blood pressure; DBP, diastolic BP; PP, pulse pressure; LVM, left ventricular mass; LVMI, left ventricular mass index. ^†^Percentage of clinic BP (< 140/90 mmHg) and 24-hour BP control (< 130/80 mmHg), *p < 0.05, **p < 0.01 versus baseline values.

At four and 13 months of follow up, one (1%) and 12 (11%) patients, respectively were receiving three or more antihypertensive drug classes. Forty-one per cent (42/103) of the patients after 13 months of treatment were receiving two or more antihypertensive drug classes.

Table 2 also shows LVM, LV wall thickness, LV internal diameters, the prevalence of LVH and the prevalence of an increased LV relative wall thickness at baseline and after four and 13 months of antihypertensive treatment. Treatment resulted in a decreased LVM and LVMI, largely through reductions in wall thickness and only minor decreases in internal diameters. Treatment produced maximal decreases in LVM, LVMI and LV wall thickness, and a maximal decrease in the proportion of patients with LVH or an increased relative wall thickness by four months of therapy, with no further changes occurring over the following nine months of therapy.

**Fig. 1:**
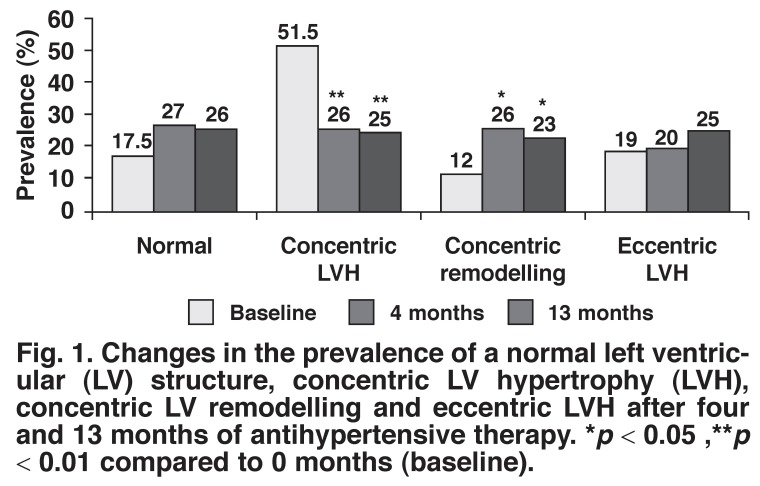
Changes in the prevalence of a normal left ventricular (LV ) structure, concentric LV hypertrophy (LV H), concentric LV remodelling and eccentric LV H after four and 13 months of antihypertensive therapy. *p < 0.05 ,**p < 0.01 compared to 0 months (baseline).

Fig. 1 shows the changes in prevalence of a normal left ventricle, concentric LVH, concentric LV remodelling and eccentric LVH after four and 13 months of antihypertensive therapy. The proportion of patients with concentric LVH decreased from 51.5 to 25% (*p* < 0.01), while the proportion with concentric LV remodelling increased from 12 to 23% (*p* < 0.05). The proportion of patients with eccentric LVH increased from 19 to 25% but this did not achieve significance. The proportion of patients with a normal left ventricle increased from 17.5 to 26% but this also failed to achieve significance.

From the 53 (51.5%) patients with concentric LVH at baseline, only seven (13%) normalised the LV structure, but 15 (28%) changed to eccentric LVH, 13 (24.5%) to concentric LV remodelling and 18 (34%) remained in the concentric LVH category after 13 months of treatment. Moreover, from 20 (19%) patients initially with eccentric LVH, only six (30%) normalised the LV structure, while four (20%) converted to concentric remodelling, and nine (45%) remained with eccentric LVH. From the 12 patients with concentric remodelling, six (50%) normalised their LV structure and five changed to eccentric LVH.

Table 3 shows the characteristics after four and 13 months of therapy of hypertensive patients, grouped according to the mass and geometry of the left ventricle. At four months of antihypertensive therapy, no significant differences were observed in age, gender, BMI, any BP measurements, BP control rates, or type of antihypertensive medication between patients grouped according to LV geometric patterns (data not shown).

**Table 3:** Characteristics Of Patients After 13 Months Of Antihypertensive Therapy Grouped According To Left Ventricular Structure

	*Geometry*			
*Geometry*	*Normal*	*Concentric LVH*	*Concentric remodelling*	*Eccentric LVH*
Number (%)	27 (26)	26 (25)	24 (23)	26 (25)
BMI (kg/m^2^)	28.9 ± 8.6	32.3 ± 5.3**	31.8 ± 5.0**	31.7 ± 5.0**
Age (years)	48.9 ± 10.1	52.3 ± 10.0	52.8 ± 8.0	51.8 ± 10.0
Female gender [*n* (%)]	22 (81)	22 (85)	20 (83)	21 (81)
24-hour SBP/DBP (mmHg)	122 ± 12/80 ± 7	128 ± 13/79 ± 7	128 ± 11/83 ± 8	128 ± 12/79 ± 9
Clinic SBP/DBP (mmHg)	129 ± 14/84 ± 8	139 ± 20/84 ± 10	135 ± 17/89 ± 9	137 ± 22/84 ± 11
24-hour BP control [n (%)]	10 (37)	14 (54)	15 (58)	9 (38)
Clinic BP control [n (%)]	19 (70)	17 (64)	19 (73)	11 (46)
24-hour PP (mmHg)	42.1 ± 8.3	49.1 ± 9.9**	45.5 ± 6.0	48.8 ± 8.5**
Clinic PP (mmHg)	44.4 ± 10.9	55.4 ± 17.3**	45.8 ± 12.2	53.3 ± 14.8**
LVM (g)	146 ± 21	208 ± 33**	148 ± 26	211 ± 36**
LVMI (g/m^2.7^)	41.2 ± 5.8	62.1 ± 8.5**	43.7 ± 5.5	63.7 ± 6.9**
LV mean wall thickness (mm)	9.3 ± 0.8	12.3 ± 1.1*	11.1 ± 1.0*	10.9 ± 1.1*
LV relative wall thickness (ratio)	0.38 ± 0.04	0.55 ± 0.08**	0.57 ± 0.07**	0.39 ± 0.05
Concentric LVH at baseline [*n* (%)]	7 (26)	18 (69)**	13 (54)	15 (58)

LV, left ventricular; LVH, left ventricular hypertrophy. SBP, systolic blood pressure; DBP, diastolic BP; PP, pulse pressure; LVM, left ventricular mass; LVMI, left ventricular mass index. *p < 0.05, **p < 0.01 compared to the normal group.

After 13 months of antihypertensive therapy no significant differences were noted in age, gender, ambulatory or clinic systolic or diastolic BP, clinic PP, BP control rates, or type of antihypertensive medication between patients grouped according to LV geometric patterns. However ambulatory and clinic PP were higher in patients with concentric and eccentric LVH.

In multiple regression analysis with age, gender, 24-hour PP and BMI included in the models, at 13 months, 24-hour (*p* = 0.02) and day PP (*p* = 0.02) were independently related to an abnormal residual LV structure after treatment (Table 4). Moreover, when baseline LVM was added to the model, 24-h PP (*p* = 0.004) still showed an independent association with residual LV structural abnormalities. Clinic PP was not a significant predictor of abnormal residual LV structural abnormalities after 13 months of therapy (*p* = 0.09). Neither 24-hour systolic/diastolic nor clinic systolic/diastolic BP control was independently associated with residual LV structural abnormalities (data not shown).

**Table 4:** Predictors Of Abnormal Geometry After 13 Months Of Therapy

	*Odds ratio*	*95% CI odds ratio*	p-*value*
Logistic regression model with 24-hour PP			
BMI (kg/m^2^)	1.087	0.985–1.198	0.096
Age (years)	1.032	0.979–1.088	0.245
Gender (female)	1.550	0.369–6.503	0.549
Baseline RWT (ratio)	0.874	0.528–1.448	0.601
24-hour PP (mmHg)	1.087	1.012–1.168	0.023
Logistic regression model with day PP			
BMI (kg/m^2^)	1.094	0.993–1.205	0.071
Age (years)	1.030	0.977–1.086	0.269
Gender (female)	1.543	0.372–6.328	0.554
Baseline RWT (ratio)	0.875	0.527–1.453	0.606
Day PP (mmHg)	1.087	1.012– 1.168	0.023

BMI, body mass index; PP, pulse pressure; RWT, relative wall thickness.

## Discussion

The main findings of this study are that in hypertensives of African ancestry, after more than a year of stepwise antihypertensive therapy, which achieved clinic BP control in 64% of patients and 24-hour BP control in 47% of patients by 13 months of therapy, the proportion of patients with a normal LV mass or geometry did not significantly increase (17.5 to 26%), while 25% remained with concentric LVH and 25% with eccentric LVH. Moreover, the proportion of patients with concentric LV remodelling increased from 12 to 23% (*p* < 0.05). These residual abnormalities in LV structure were noted even though therapy resulted in a decrease in LVM and relative wall thickness.

Although a number of studies have demonstrated that antihypertensive therapy can regress LVH as well as reduce LV relative wall thickness, few studies have reported on the ability of therapy to normalise LV structure. In this regard, in contrast to the outcome of the present study where 25% of patients remained with concentric LVH and only 26% had a normal LV structure after one year of therapy, in the LIFE study after one year of therapy in 853 patients with electrocardiographic LVH at baseline, the prevalence of residual concentric LVH was 6% and the proportion of patients with a normal LV structure was 51%.^13^ In another study conducted in 182 patients, 30% of whom had concentric LVH at baseline, only 14% of 182 patients had concentric LVH after one year of therapy.^14^

An explanation for the markedly higher prevalence of residual concentric LVH and lower prevalence of a normal LV structure after one year of antihypertensive therapy in our study compared to previous studies^13^,^14^ is most likely the higher proportion of patients with an increased baseline concentric LVH. Indeed, in previous studies,^13^,^14^ the prevalence of concentric LVH at baseline was approximately 10 to 30%, compared to the approximately 51% of patients with concentric LVH in our study. Moreover, in previous studies^13^,^14^ mean baseline LV relative wall thickness ranged from 0.41 to 0.45, while in the present study, mean baseline LV relative wall thickness was 0.50.

The markedly higher prevalence of concentric LVH (> 50%) and mean relative wall thickness values noted in our study, which was conducted in a group of patients of African descent, compared to previous studies conducted in patients of European descent,^13^,^14^ is consistent with ethnic disparities in the extent of concentric LV remodelling previously reported to exist between groups of African and European descent.^21^ Furthermore, the markedly higher prevalence of concentric LVH and mean relative wall thickness values noted in the present compared to previous studies^13^,^14^ is consistent with the high prevalence of obesity in our sample. In this regard, we have recently demonstrated a strong relationship between central obesity and concentric LVH and remodelling in patients of African descent.^26^

The high prevalence of a residual abnormality in LV structure even after a year of antihypertensive therapy in the present study is not explained by poor systolic and diastolic BP control. Neither clinic nor 24 hour systolic/diastolic BP and clinic BP control was independently associated with residual LV structural abnormalities. Indeed, in the present study the clinic BP control rates were 64% compared to 60% in previous studies reporting on a low prevalence of residual LV structural abnormalities after one year of therapy.^14^

Moreover, unlike in other studies,^13^,^14^ we used ambulatory BP monitoring to further evaluate the effect of therapy on 24-hour BP profiles. In this regard, 47% of patients achieved 24-hour BP values below 130/80 mmHg. Importantly however, in a separate model, 24-hour PP was independently related to an abnormal LV structure. In contrast, office PP could not account for the variability in left ventricular mass or relative wall thickness in the LIFE study after one year of antihypertensive treatment.^13^ Similarly, we were unable to show a relationship between clinic PP and residual LV structural changes.

The high prevalence of a residual abnormality in LV structure even after a year of antihypertensive therapy in the present study is not explained by a poor response of the left ventricle to therapy. Indeed, in the present study, the mean decrease in LV relative wall thickness produced by antihypertensive therapy was similar to that noted in other studies reporting on a low prevalence of residual LV structural abnormalities after a year of therapy.^13^,^14^ Although the present study was not designed to assess the effects of specific drug classes, we were also unable to identify a relationship between drug class and the LV response to therapy.

Importantly, of the 51.5% of patients with concentric LVH at baseline, 28% changed their LV geometric pattern to eccentric LVH, and 24.5% normalised their LVMI but maintained a concentric LV geometry, while 34% remained in the concentric LVH category after 13 months of treatment. Therefore, although approximately half of these patients no longer remained in the concentric LVH category, only 13% normalised their geometry after a year of therapy. These data suggest that, at least in our population sample, concentric LVH is difficult to normalise. In this regard, one previous study conducted in patients of European descent has demonstrated similar outcomes where only 27% of those with concentric LVH at baseline normalised LV structure.^14^ In contrast however, in the LIFE study, 75% of patients with concentric LVH at baseline normalised LV structure after one year of therapy.^13^ A reason for these discrepancies is not apparent and further studies are required to address this issue.

The limitations of the present study include the following. A large proportion of the study group consisted of females and hence the outcomes may be specific to females. Second, the patients participating in the trial were recruited from an urban African community with a high unemployment rate, and a number of participants relocated to alternate areas where employment was available. Therefore retention rates at 13 months were low.^23^ Third, antihypertensive treatment was based on drug combinations from a variety of classes to achieve BP control and therefore we cannot draw conclusions as to whether the effect noted in our study applies to all antihypertensive agents. However, the present approach is in line with what is likely to occur in clinical practice where combination therapy with different classes of agents is the most likely method of achieving BP control. Lastly, we did not assess the effect of treatment beyond 13 months and hence we do not know what antihypertensive therapy for two to three years may have achieved. However, we do not believe that further significant changes in LV structure would have occurred, as maximal changes were already present at four months of therapy and no further changes were noted from four to 13 months of therapy.

## Conclusion

In this study we show that after more than a year of antihypertensive therapy in hypertensives of African ancestry, including combination therapy where required, no significant change in the proportion of patients with a normal left ventricle occurred. Twenty-five per cent still had residual concentric LVH, and a further 25% eccentric LVH, while the proportion with concentric LV remodelling increased. Neither clinic systolic/diastolic nor 24-hour systolic/diastolic BP control accounted for these residual changes. Twenty-four-hour and day PP were associated with residual LV structural changes. These findings suggest that optimal therapeutic strategies for reducing cardiovascular risk in hypertensives of African ancestry have yet to be achieved. Further studies are required to evaluate whether reducing PP to acceptable target levels would improve the capacity to reverse LV structural abnormalities.
